# Synthesis and In Vitro/Ex Vivo Characterizations of Ceftriaxone-Loaded Sodium Alginate/poly(vinyl alcohol) Clay Reinforced Nanocomposites: Possible Applications in Wound Healing

**DOI:** 10.3390/ma15113885

**Published:** 2022-05-30

**Authors:** Shabana Bibi, Sadullah Mir, Wajid Rehman, Farid Menaa, Alia Gul, Fatima Saad Salem Alaryani, Ali M. Alqahtani, Sirajul Haq, Magda H. Abdellatif

**Affiliations:** 1 Department of Chemistry, Hazara University, Mansehra 21220, Pakistan; sjamil604@gmail.com; 2 Department of Chemistry, COMSATS University Islamabad, Abbottabad Campus, Islamabad 22060, Pakistan; sadullah@cuiatd.edu.pk; 3 Departments of Internal Medicine and Nanomedicine, California Innovations Corporation, San Diego, CA 92037, USA; 4 Department of Botany, Hazara University, Mansehra 21220, Pakistan; aliagul@hu.edu.pk; 5 Department of Biology, Faculty of Sciences, University of Jeddah, Jeddah 21959, Saudi Arabia; fsalaryani@uj.edu.sa; 6 Department of Pharmacology, College of Pharmacy, King Khalid University, Guraiger, Abha 62529, Saudi Arabia; amsfr@kku.edu.sa; 7 Department of Chemistry, University of Azad Jammu & Kashmir, Muzaffarabad 13100, Pakistan; cii_raj@yahoo.com; 8 Department of Chemistry, College of Sciences, Taif University, Taif 21944, Saudi Arabia; m.hasan@tu.edu.sa

**Keywords:** sodium alginate, PVA, ceftriaxone, drug delivery, nanoclay, nanomaterials

## Abstract

(1) Background: Nanocomposite films are widely applied in the pharmaceutical industry (e.g., nanodrug delivery systems—NDDS). Indeed, these nanomaterials can be produced at a large industrial scale and display valuable properties (e.g., antibacterial, renewability, biodegradability, bioavailability, safety, tissue-specific targeting, and biocompatibility), which can enhance the activity of conventional marketed drugs. (2) Aim: To fabricate and investigate the in vitro properties of the antibiotic ceftriaxone sodium (CTX) once encapsulated into sodium alginate (SA)/poly(vinyl alcohol)PVA-clay reinforced nanocomposite films. (3) Methods: Different ratios of the polymers (i.e., SA, PVA) and CTX drug were used for the synthesis of nanocomposite films by solvent casting technique. Montmorillonite (MMT), modified organically, was added as a nanofiller to increase their thermal and mechanical strength. The prepared samples were physically characterized by thermogravimetric analysis (TGA), X-ray diffraction (XRD), scanning electronic microscopy (SEM), and energy-dispersive X-ray analysis (EDX). The physicochemical behavior (i.e., swelling, erosion, dissolution/drug release behavior and rat skin permeation) was also assessed. Comparisons were made with the currently marketed free CTX dosage form. (4) Results: TGA of the nanoformulation showed increased thermostability. XRD revealed its semi-crystalline nature. SEM depicted a homogeneous drug-loaded SA/PVA nanocomposite with an average size ranging between 300 and 500 nm. EDX confirmed the elemental composition and uniform distribution of mixing components. The water entrapment efficiency study showed that the highest swelling and erosion ratio is encountered with the nanoformulations S100(3) and S100D15(3). Ex vivo permeation revealed a bi-step discharge mode with an early burst liberation chased by continued drug discharge of devised nanoparticles (NPs). The dissolution studies of the drug-loaded polymer nanocomposites elicited sustained pH-dependent drug release. The cumulative drug release was the highest (90.93%) with S100D15(3). (5) Conclusion: S100D15(3) was the finest formulation. To the best of our knowledge, we also pioneered the use of solvent casting for the preparation of such nanoformulations. Polymers and reinforcing agent, concentrations and pH were rate-deterring features for the preparation of the optimized formulation. Thus, CTX-loaded SA/PVA-MMT reinforced nanocomposite appeared as a promising nanodrug delivery system (NDDS) based on its in vitro physicochemical properties.

## 1. Introduction

The history of pharmacy is as old as human history; both operate analogously to each other. Man was able to discover the treatments for complex illnesses that he has gained thus far due to his research expertise, affection, practical experiences, and judgment.

Nowadays, special focus is placed on using polymers to make polystyrene vials, rubber closures, tubes for sets of injections, and PVA-based elastic bags for storing blood and intravenous solutions. Previously, polymers were commonly restricted to packaging instead of drug delivery systems (DDS). Consequently, the unification of pharmaceutics and polymer sciences results from the incorporation of polymers in the devising and advancement of DDS [1]. Thereby, targeted drug delivery was shown to reduce systemic toxicity by carrying the drug efficiently from dosage form to targeted organ according to the necessitation of therapeutic level without the drug loss [2].

As drugs are generally administered through the oral route, importance is placed on the advancement and growth of controlled oral DDS. Drugs are enthusiastically taken in and eliminated due to their hydrophilic nature and short half-life (2–3 h) [3]. Drugs are administered recurrently to minimize patient acquiescence, as over-doses are harmful due to drug deposition [4]. However, it is worth mentioning that about 95% of the latest possible remedial drugs have extremely low pharmacokinetics and biopharmaceutical effects [5].

This demands far-reaching research to lessen the harmful effects of these drugs while enhancing their efficacy by formulating original polymer-based systems for sustained drug delivery.

Over the last few years, nanotechnology has made significant advances in many disciplines [6], including pharmacy [7]. Nanomedicines are nanocarriers that play a major role in site-directing the drug without influencing the healthy tissues. The high efficiency of nano-sized drugs has lessened dosages and safety concerns while expanding the therapeutic index [7]. Polymers of natural (e.g., polysaccharides like sodium alginate or chitosan) and/or of synthetic origin (e.g., PVA), when unified with NPs [e.g., MMT organo-clays, carbon nanofibers (CNFs), carbon nanotubes (CNTs), nano-silica (N-silica), nano-aluminum oxide (Al_2_O_3_), nano-titanium oxide (TiO_2_)], produce polymer nanocomposites [8]. 

Natural clay minerals are very appropriate for producing customized DDS due to their protuberance dimensions, crystalline conformation, elevated specific surface area, fast interchange of cations, charge, and dimension of colloidal units [9]. The minerals are assimilated inside the polymer acting as a host carrier to command the dissemination ratio of the scattered slow-release substance (e.g., drug). NPs lessen the penetrability of the polymers or block the dispersal of functional stuff which is being discharged by expanding the size of the dissemination track; the discharge rate of slow-release polymers is also decelerated more by clay [10].

However, enhancing the properties of polymer nanocomposites is a continuous challenge for their widespread use in research [11]. Such biomaterials already have applications in wound healing, gene therapy, tissue engineering, and controlled drug delivery applications [12]. The tremendous interest and progress in polymer nanocomposites is due to their biodegradability, non-toxicity, biocompatibility, and environmental susceptibility [13].

Polysaccharides (e.g., SA) are universally used in drug delivery functions due to their bioavailability and reproducibility. They are biodegradable, biocompatible, and possess a low immunogenic response. Consequently, they are considered good candidates for drug delivery applications in response to physiological stimuli [14]. Therefore, for almost two decades, research on the preparation of alginate-based NPs has been gradually increasing in drug development [15] and in genetic engineering, with a significant potential for gene transfer [16]. Interestingly, alginate can be blended with chitosan (CS) for drug delivery applications [17]. Similarly, Demiroz et al. used alginate for producing mesalazine tablets for drug delivery in the intestine [18].

PVA has been used in polymer science and technology research for over 50 years. This synthetic semi-crystalline polymer is produced in the largest volume today via hydrolysis of polyvinyl acetate (PVAc) on an industrial scale; it displays many valuable properties for wound care and topical applications (e.g., high biodegradability, excellent hydrophilicity, high biocompatibility, non-toxicity, excellent thermal stability, excellent processability, and excellent film forming ability). To be used in medical and pharmaceutical applications, PVA should be cross-linked or stabilized using physical entanglement which help to overcome the aging effect. PVA has often been blended with other polymers (e.g., gelatin, SA) for wound care and drug delivery applications [19]. The goal of such blends is generally to prepare new material with tailor-made properties, improve the processability, modify physical properties, and reduce the cost [19].

Among blended polymers, Kim et al. cross-linked SA/PVA polymers by freeze–thawing and encapsulated nitrofurazone in the prepared SA/PVA hydrogel for its use as a wound dressing material [19]. Chen et al. reported the encapsulation of newly synthetized tea polyphenol nanospheres in SA/PVA hydrogels for promoting wound healing and regulating the immune response in a diabetic rat model [20]. Similarly, the study led by Han et al. indicated that electrospun lutein-loaded SA/PVA nanofibers (NFs) show great potential as a controlled release system [21].

CTX is a third-generation cephalosporin, a cell wall synthesis inhibitor, and a broad-spectrum beta-lactam antibiotic, and was first released in 1982 for the treatment of severe infections including those caused by multi-drug-resistant (MDR) strains [22]. This antibiotic was commonly prescribed, because of its performance and low toxicity, for respiratory bacterial infections (bronchitis, pneumonia) and urinary tract infections [22]. Nevertheless, due to the ever-increasing emergence and spread of antimicrobial drug resistance (AMR), including CTX resistance by virtue to its extensive use over the past decade, several promising nanotechnology-based delivery strategies (e.g., through polymeric films and/or lipid nanocarriers) have been initiated [23].

**Scheme 1:**
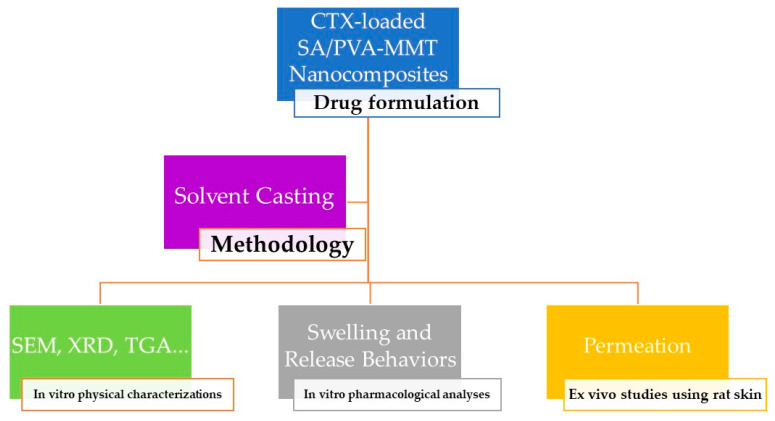
CTX-loaded and unloaded SA/PVA-MMT nanocomposite films.

In the present paper, CTX-loaded SA/PVA-MMT nanocomposite films were prepared using the solvent casting technique (Scheme 1). The physical characteristics of these formulations were characterized in vitro by SEM, EDX, XRD, and TGA. Their potential pharmaceutical applications were investigated by evaluating the in vitro swelling and erosion ratio, as well as the in vitro cumulative drug release and ex vivo permeation. 

## 2. Materials and Methods

### 2.1. Reagents

SA was purchased from the International Laboratory (USA). PVA was obtained from Daejung (Siheung-si, Korea). Organically modified MMT was provided by Sigma Aldrich (Saint Louis, MO, USA) containing 25–30 weight% of trimethylstearyl ammonium. Glycerol with a purity of 84–88% was obtained from Riedel-de Haen (Seelze, Germany). The CTX drug was gifted by Global Pharmaceuticals Industry Islamabad (Islamabad Capital Territory, Pakistan). Sodium hydroxide (purity of 98–99.99%), potassium chloride (purity > 99.0%), Hydrochloric acid (HCl) (purity of ≥99.855%), and glacial acetic acid (purity of ≥99.855%) was purchased from Sigma Aldrich (St. Louis, MI, USA). Methanol, ethanol, chloroform, sodium acetate (NaOAc), and mono-potassium phosphate (KH_2_PO_4_) were provided by Merck and Co. (Darmstadt, Germany). Distilled water (dH_2_O) was provided by COMSATS University Islamabad, Abbottabad Campus (Islamabad, Pakistan).

### 2.2. Preparation of SA/PVA Nanocomposites

Polymer nanocomposites were prepared by solvent casting technique [24,25]. First, aqueous solutions of SA and PVA were prepared by dissolving them separately in beakers filled with 50 mL of distilled water; glycerol was used as a plasticizer. After their dissolution in water, SA and PVA were mixed in 1:1 and 1:0 ratios to prepare different formulations and placed on a hot plate for constant heating at 60 °C for 30 min. The mixture was stirred continuously using a mechanical stirrer. Next, 3 g of nano-clay was put into the polymer mixture as reinforcement, and after 15 min, 15 g CTX drug was dissolved in 30 mL distilled water. A few drops of glycerol (about 50 g) were also incorporated with continuous stirring at 60 °C. At constant temperature, the entire solution was stirred for 30 min. After full dissolution, the final product (i.e., drug-loaded PVA/SA clay reinforced nanocarrier) was put into petri dishes and kept in an oven at 50 °C for 24 h to achieve absolute drying. After drying, solid, even, plastic-type films were created. Six varied films were prepared by varying the quantities of mixing polymers (P and/or S) and drug (D) (Table 1). Clay (M) was kept as constant (3 g). Consequently, codes were allotted as S100(3), P100(3), S50P50(3), S100D15(3), P100D15(3), and S50P50D15(3).

### 2.3. Physico-Chemical Characterizations

#### 2.3.1. Thermogravimetric Analysis

The thermal stability of the drug-loaded (and unloaded) SA/PVA nanocomposites reinforced by MMT nanoclay was evaluated by thermogravimetric analysis (TGA) [26] using a Mettler Tolledo TGA/DSC Star 1 system with an Alumina 70 μL pan. TGA examines the relationship between one or more predictors (independent variables) and a response variable (dependent variable), with the goal of defining a “best fit” model of the relationship [22]. TGA has allowed us to measure temperature-induced mass changes and confirm the CTX loading into the prepared MMT reinforced-polymeric nanocomposite. TGA scans were carried out at a heating rate of 10 °C/min under nitrogen atmosphere at the purge rate of 30 mL/min, from 25 to 600 °C. The curve fitting was performed using “Origin Pro 8.0”. The following parameters were reported: (i) the temperature corresponding to 10% weight loss (WL T_10%_), (ii) the temperature at maximum decomposition rate (T_max_), (iii) the weight loss associated with T_max_(WL T_max_), and (iv) the residual weight (RW) associated with maximum WL (WL_max_).

#### 2.3.2. XRD Analysis

To find out the amorphous and crystalline nature of drug-loaded (and unloaded) SA/PVA nanocomposites reinforced by MMT nanoclay, XRD analysis [26,27] was carried out at 2θ range of 10–90° using Philips Panalytical XPERT PRO 3040/60 (CAE Silicon Valley trading operation, Santa Clara, CA, USA).

#### 2.3.3. SEM and EDX Analyses

High-resolution imaging of the surface morphology and microstructure of the synthesized samples was evaluated using SEM [27,28,29]. Therefore, loaded SA/PVA nanocomposites reinforced by MMT nanoclay were subjected to SEM measurements comparatively to blank/unloaded SA/PVA nanocomposites reinforced by MMT nanoclay (control). Samples of 1/1 cm were placed on the copper holder using a sticky carbon tap, and the thin gold layer was coated on it using a gold stammer following a previously published method. This coating is commonly required to obtain a clear micrograph of an insulating material, although it is so thin (200 Å) that it does not hinder the identification of specific minerals. Their morphologies, including average diameters, were then assessed under a SEM (TESCAN MIRA3) operating at the acceleration voltage of 0–20 kV. The image analysis program Image J (US National Institute of Health, Bethesda, MD, USA), which uses grayscale level processing based on image structure, was used to characterize the SEM micrographs in the original magnification of 25×. Under the same conditions, EDX helped to determine the matrix’s elemental composition and mixing components’ purity.

### 2.4. Swelling Test

The six prepared formulations (i.e., S100(3), P100(3), S50P50(3), S100D15(3), P100D15(3), and S50P50D15(3)) were examined for swelling capacity [23,30,31] at room temperature (RT) with three different buffers of pH 1.2 (HCl solution), pH 4.5 (sodium acetate buffer), and pH 6.8 (phosphate buffer saline, 1× PBS). Next, 30 mg of each film was immersed separately in Petri dishes containing 10 mL of each buffer. The films could swell for a specific time in the buffer solutions. Swelling weights were recorded on an analytical balance with time intervals of 1, 2, 3, 4, and 5 min. At each interval of time, the nanocomposite films were removed from the buffer, wiped with a tissue paper to clear the extra buffer, and was weighed to measure the degree of swelling.

The equilibrium swelling ratio (SR) of each film was then calculated using the following formula:

(1) $$\mathrm{SR}=\frac{\mathrm{Ws}}{\mathrm{Wd}}$$

where, W_S_ = the weight of the swollen film and W_d_ = the dry weight of the film (30 mg). 

The water content (WC) was measured by applying the following formula:

WC (%) = [W_S_ − W_d_/W_S_] × 100 (2)

where, W_S_ = the weight of the swollen film, and W_d_ = the dry weight of the film (30 mg).

The experiment was performed in triplicate. Mean value and standard deviation have been mentioned in the graph.

### 2.5. Erosion Test

After the swelling test, the wet films were kept in the open air for drying. The dried films were then weighed individually at various time gaps until a steady weight was obtained. This test was triplicated. The erosion ratio (ER) of each film was then determined using the following formula [31].

ES (%) = [W_S_ − W*_f_*/W_S_] × 100 (3)

where, W_S_ = the weight of the swollen film and W*_f_* = the final dry weight.

### 2.6. Drug Content Uniformity Test

A drug content uniformity test [27] was carried out to examine the uniform division/distribution of the drug in attained nanocomposite films. For this purpose, 30 mg of each film was crushed and placed in 10 mL of 1× PBS (pH 6.8). This solution was stirred continuously for about 1 h to 2 h. The resulting solutions were then filtered. 2 mL of this filtrate was shifted in a volumetric flask of 50 mL and the volume was increased to the mark using 1× PBS solution (pH 6.8). To ensure uniformity of the solution, the flask was shaken continuously for about 15 min. The experiment was performed in triplicate, and the absorbance was measured at 323 nm by UV-Vis spectrophotometry (Shimadzu UV-2600 Spectrometer, Kyoto, Japan).

### 2.7. Calibration Curve Plot

The standard calibration curve was plotted to get the linear equation of CTX, based on a previously published method [32]. Briefly, 100 mg of the drug was dissolved in 100 mL of KH_2_PO_4_ buffer (pH 6.8). Then, a set of dilution was prepared in the range of 2–20 μg/mL in the same buffer. These dilutions were eventually analyzed by UV/Vis spectrophotometer at 323 nm by UV-Vis spectrophotometry (Shimadzu UV-2600 Spectrometer, Kyoto, Japan) by taking 1× PBS (pH 6.8) as blank. With the help of the obtained linear equation, the concentration of CTX could be calculated.

### 2.8. Ex Vivo Permeation Test

A CTX permeation study was performed through the rat skin using a Franz Diffusion Cell, following on previously published methods [8,33,34]. 

#### 2.8.1. Preparation of Rat Skin

Seven Sprague–Dawley rats weighing 270 g and aged 6–8 months were used in the permeation study. These rats were provided by the animal house Department of Pharmacy, COMSATS Abbottabad, Pakistan. Chloroform was used as a sedative to anaesthetize the rats. The skin was shaved using electrical and hand razor. After that, belly skin and underlying fat were removed using a surgical blade and scissors. The skin was then soaked in 0.9% NaCl regular salt solution for 120 min to get rid of seeping out enzymes and other dermal debris. Afterwards, the skin was thoroughly washed out with an excess of water, wrapped in aluminum foil, and was kept at −20 °C in a freezer until it was used. Just before the experiment, the skin was defrosted at RT before use. 

#### 2.8.2. Set Up of Franz Diffusion Cell

The Franz diffusion cell was comprised of a receptor part and a donor part. The prepared rat skin was mounted in the center of the receptor and donor compartments of the Franz diffusion cells. The skin was placed in such a way that the dermal portion of the skin was set toward the receptor portion while the stratum corneum part lay towards the donor portion of Franz cells. The skin was clamped using clips in between the two compartments of Franz cells. The receptor part of the Franz diffusion cell was filled with about 4.5 mL of 1× PBS with pH 6.8. These Franz cells were placed in a water bath with a constant temperature maintained at 37 °C. Next, 40 mg of CTX drug-loaded nanocomposite film was placed in the donor compartment of the Franz cell for estimation of the drug permeation, and 1× PBS inside the receptor compartment was continuously stirred using small magnetic stirring bars. After a regular period of 1, 2, 3, 4, 5, 6, and 24 h, about 200 µL buffer was withdrawn by micropipette from the receptor portion and was analyzed at 323 nm by UV-Vis spectrophotometry (Shimadzu UV-2600 Spectrometer, Kyoto, Japan) to get the concentration of CTX permeated through the rat skin. The withdrawn amount of buffer was replaced regularly each time the reading was made.

### 2.9. Dissolution Study/Drug Release Behavior

Dissolution testing is a requirement for all solid oral dosage forms and is widely used throughout the development life-cycle for evaluating the drug release characteristics and consistency/stability of a pharmaceutical product [22,28,29,34]. To study the dissolution of the CTX-loaded polymer nanocomposite, 25 mg of films was kept in a beaker containing 100 mL of 1× PBS at 37 °C. After every 10 min, 5 mL of the sample was collected, and the liquor was refilled by adding 1× PBS (pH 6.8) up to the mark. The release of the drug was determined using UV-VIS spectrophotometry at 323 nm. 1× PBS of pH 6.8 was taken as the reference standard.

### 2.10. Statistical Analysis

All experiments were at least triplicated. All data are expressed as the mean ± standard deviation (SD). The statistical analysis of differences was performed using a t-test in OriginPro 2018. *p* < 0.05 indicates a statistically significant difference, while *p* > 0.05 means statistical insignificance

## 3. Results 

### 3.1. Thermogravimetric Analysis (TGA)

**Figure 1:**
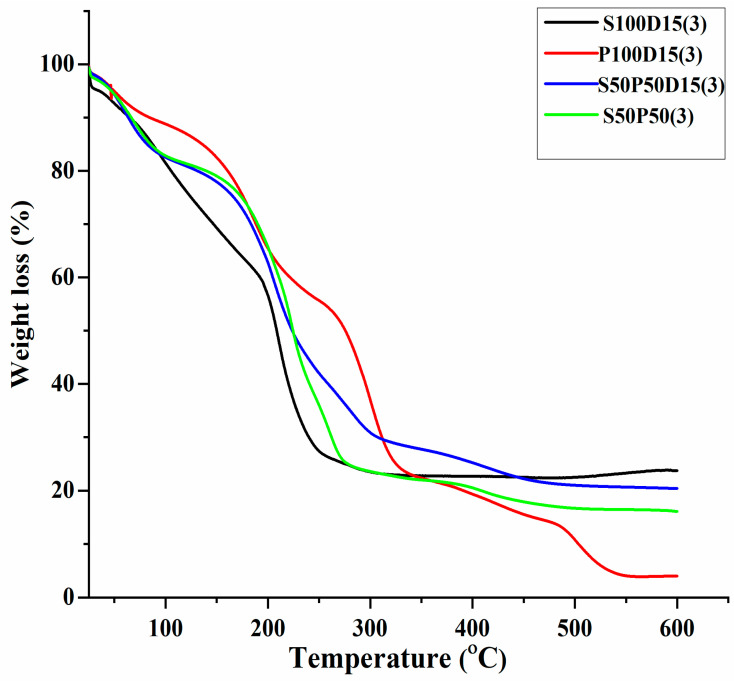
TGA thermograms of drug loaded (and unloaded) SA/PVA-MMT clay reinforced nanocomposite films.

TGA showed the strength of samples over increasing temperatures (Figure 1). Their respective percentages of mass loss at various temperatures are indicated in Table 2.

In all four tested samples (i.e., S50P50(3), S100D15(3), P100D15(3), and S50P50D15(3)), the mass loss occurred in three distinct stages [35]. In the first stage, nearly 15–24% of the mass loss occurred at 120 °C, which is due to the evaporation of absorbed water. At the second stage, about 43.3% of the mass is lost at 200 °C, which is due to a complex degradation process of polysaccharide rings. After this stage, mass gradually decreases with a maximum loss of 80% that occurred at 400 °C, most likely due to the dehydration and decomposition of the SA backbone that took place in S50P50(3), S100D15(3), and S50P50D15(3). P100D15(3) showed the least stability at 400 °C, losing mass of 81%. Side chain decomposition of PVA started up to 200 °C which showed the elimination of amorphous part of PVA. After this, at 400 °C, a maximum mass loss of 81% was due to the backbone decomposition of PVA. In the temperature range of 475–482 °C, formation of anhydride chains and the destruction of the cross-linked network took place, after which 4.02% residue is left; this is the minimum value in this series. As temperature further increased from 588 °C, onward SA is converted into sodium carbonate and residue of carbonized material. At 600 °C maximum residue, 23.8% of S100D15(3) is left, which is the maximum in this series. 

So, it has been seen that in the temperature range of 25–200 °C, the maximum stability is shown by S50P50(3) while the least stable sample is S100D15(3). When the temperature was increased to the range of 200–400 °C, an onward thermal stability was shown by sample S50P50D15(3) containing SA and PVA drug, but in the temperature range of 400–600 °C, S100D15(3) is most stable and remains stable beyond this temperature, because the maximum residue is left by this sample. Formulation S100D15(3) contains a high amount of SA, while formulation P100D15(3) contains 100% PVA and is least stable. The residue percentage showed that clay and drug increased the thermal stability of SA more than PVA. Previous studies revealed that the addition of nanoclay increases the thermal stability of nanocomposites [36].

Overall, in CTX-loaded samples S100D15(3), P100D15(3), and S50P50D15(3), it has been observed that in the temperature range of 25 °C to 400 °C, S100D15(3) had the highest thermal stability, while in the temperature range of 200 °C to 400 °C, P100D15(3) had the least thermal stability. At 120 °C, the maximum water loss is shown by S100D15(3). The water loss of P100D15(3) is the least until 120 °C, where the highest concentration of PVA and an increased interaction between polymer and water molecules are observed. The result is the slowing of water evaporation. All the three formulations showed a second mass loss at 200 °C. At this temperature, S100D15(3), P100D15(3), and S50P50D15(3) showed 43.3%, 34.5%, and 37.2% mass loss, respectively. This showed that the thermal stability is least for S100D15(3) at this point. Then, a gradual decrease in mass appeared in all formulations from 200–400 °C. At 400 °C, P100D15(3) showed the maximum mass loss of 81%, whereas a minimum mass (74.8%) is lost by S50P50D15(3). Eventually, at 600 °C, the maximum residue (23.8%) is left by S100D15(3) and the minimum residue (4.02%) by P100D15(3). The maximum residue is left by S100D15(3). Therefore, it can be concluded that the addition of drug and an increase in its concentration stabilized the nanocomposite.

### 3.2. X-ray Diffraction Analysis

**Figure 2:**
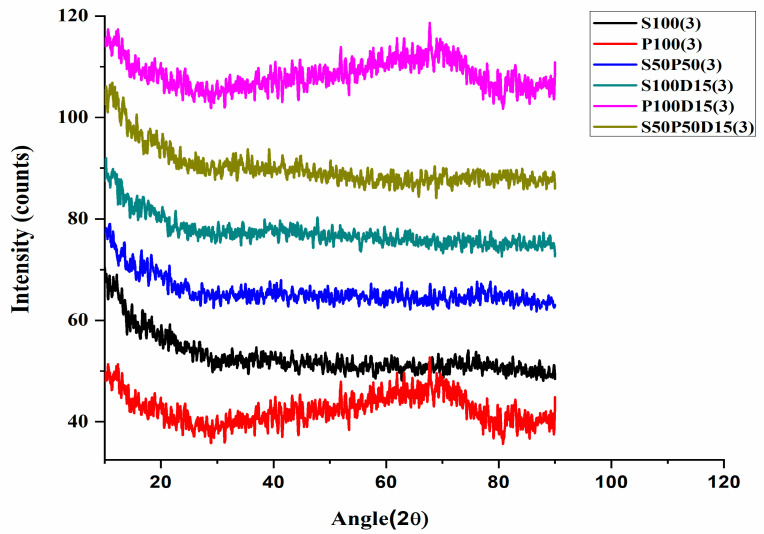
XRD patterns of drug-loaded (and unloaded) SA/PVA-MMT clay reinforced nanocomposite films.

XRD analysis was conducted to determine the crystallinity and/or amorphous nature of nanocomposite films by varying the SA/PVA ratio. The XRD pattern of nanocomposite films is shown in Figure 2. 

In all the unloaded drug samples, S100(3), P100(3), and S50P50(3), the ratio of SA and PVA is different while the amount of clay is constant (i.e., 3%). In these samples, the drug was absent. XRD studies revealed that SA is amorphous [37], since there was no sharp diffraction peak, while PVA has a crystalline nature with two characteristic peaks at 2θ= 20° and 2θ = 40° [38]. In all samples, characteristic peaks at 2θ = 10.6 and 2θ =19.7 appeared with d-spacing of 8.3 and 4.5, respectively. All other peaks in samples are due to the presence of MMT and its interaction with polymers. Sharp peaks in the sample S100(3) appeared, as it contains only SA and organically modified MMT, so the crystalline nature of this sample may be due to the presence of MMT, as it is highly crystalline. MMT gives a sharp peak at 2θ= 8.5°, but this peak is shifted towards the lower 2θ value; this is explained by the intercalation of polymer into clay sheets [39]. It also gives broad peaks in the range of 2θ = 5–6°. In an analogous way, the formulations S100(3), S50P50(3), S100D15(3), and S50P50D15(3) gave broad peaks at 2θ = 19.7°, 2θ = 19.5°, and 2θ = 20.8°. The broad peak is observed because of the amorphous nature of SA, while formulations S50P50(3) and S50P50D15(3) consist of PVA in addition to SA and MMT clay. PVA showed sharp characteristic peaks at 19.5°, 22.7°, and 40.6° due to the crystalline character and strong H-bonding between hydroxyl (-OH) groups. The slight variations in the 2θ values of PVA and SA peaks were due to the semi-crystalline nature of blends. The peak at 2θ = 13.73° is from SA, which consistently appears in all the samples except P100(3) and P100D15(3). The second sample, P100(3), showed two peaks of PVA at 2θ = 22° and 2θ = 41°. This sample had less intense peaks as compared to sample S100(3), which may be attributed to the fact that PVA underwent exfoliation with MMT or a weak hydrogen bonding was formed between clay and PVA functional groups which decreased the interplanar spacing and less intense peaks were observed [39,40]. XRD results showed the possible interaction of SA and PVA by making H-bond between the hydroxyl group of PVA and carboxyl or the hydroxyl group in SA which might hinder the crystalline structure of SA by making strong molecular interaction. In all the XRD patterns of SA/PVA, 2θ = 19.6° was observed, and the peaks of pure SA and PVA were absent. This amorphous behavior resulted from weak interactions produced due to the blending of polymers. If the polymers were not blended properly, the respective peaks of both the polymers would have appeared separately.

In all the loaded drug samples, S100D15(3), P100D15(3), and S50P50D15(3), the drug showed intense peaks at 2θ = 19°, 2θ = 20°, and 2θ = 21°, as it has a crystalline nature. Two characteristic peaks at 2θ = 10.6° and 2θ = 19.7° appeared in all these samples. According to previous studies, SA gives sharp peaks at 2θ= 6.55°, 2θ= 6.49°, 2θ= 13.6°, 2θ= 14°, and 2θ= 23° [37], while PVA showed its crystalline nature with two characteristic peaks at 2θ = 20° and 2θ = 40° [38]. MMT elicited peaks at 2θ = 6.98°, 2θ = 8.5°, 2θ = 19.6°, 2θ = 20.3°, 2θ = 61.8°, 2θ = 21.3°, and 2θ = 35.5°, while pure CTX spectral pattern exhibited peaks at 2θ = 11–13°, 2θ= 18–25°, and 2θ = 28°. The reported values indicated that all the components gave a peak at 2θ = 20° which makes it complex in the separation of different peaks from each other except SA at 23°. In all samples, an intense broad peak around 2θ = 20° was seen which confirmed the presence of these components in the semi-crystalline form in nanocomposites. CTX is known to elicit a crystalline structure [41]; many sharp peaks are displayed by its pure form at 2θ = 11–13°, at 2θ = 18–25°, and at 2θ = 28°. The drug-loaded samples S100D15(3), P100D15(3), and S50P50D15(3) showed a peak between at 2θ = 11–13° and at 2θ = 22–25°, which confirmed that adding the drug has increased the crystallinity of nanocomposites. In these samples, the drug peaks were decreased in intensity as compared to pure sample, which revealed the drug interaction with the polymers, resulting in changes in atomic densities in a specific plane of the crystal lattice [41,42].

### 3.3. SEM Analysis

**Figure 3:**
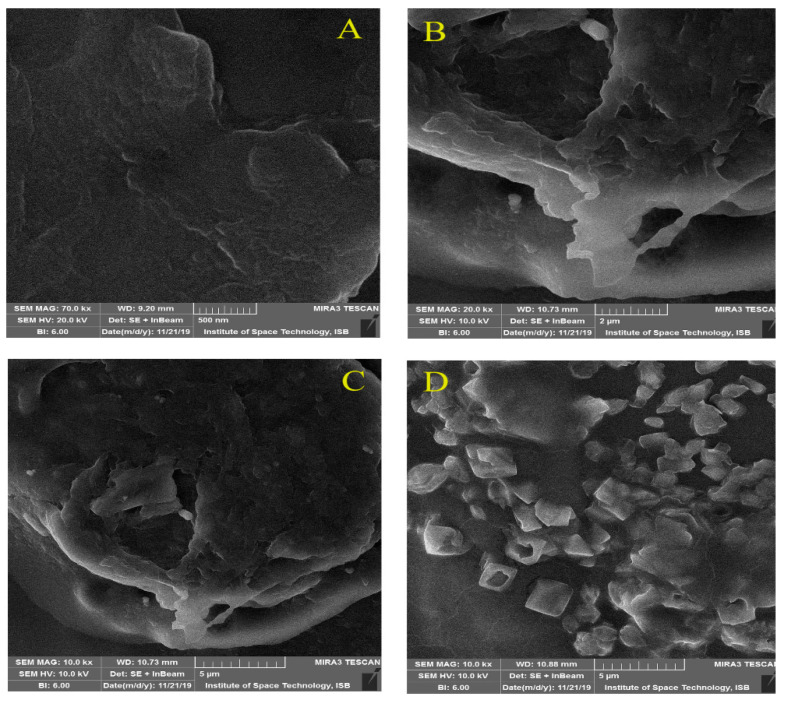
SEM of (**A**,**B**) S50P50 (**C**,**D**) S50P50D15(3). Bar scales are indicated.

Figure 3A,B showed SEM micrographs of S50P50(3) at two distinct magnifications, and Figure 3C,D showed SEM micrographs of S50P50D15(3) at the same magnification, but with two different morphologies due to the non-uniform drug adsorption at the surface. This study was carried out to investigate the morphology and compatibility among the (SA, PVA) polymers, MMT, and CTX drug. 

Figure 3A,B depict surface properties of S50P50(3). A dark background represents the consistency in the matrix, while nanoclay is dispersed equivalently in the entire matrix in the form of tiny chips. SEM image of S50P50(3) inferred the presence of voids in the films generating a highly porous structure, which is responsible for the hydrophilic behavior of films as water penetrates through these voids into the polymeric structure.

Figure 3C,D showed large blocks; nanoclay has made small dot-like structures which are consistently dispersed in the formulation proving its high compatibility with the matrix. The presence of drug in the form of large-sized blocks or long rod-shaped crystals confirmed its crystalline structure, which was also revealed by previous studies [41].

The average particle size (PS) of S50P50(3) and S50P50D15 formulations ranged from 300 nm to 500 nm.

Taken together, the SEM micrographs of S50P50(3) and S50P50D15(3) showed that all components are homogeneously dispersed in a polymer matrix.

All the formulations contained SA, PVA, and MMT clay. The pure SA revealed soft and consistent morphology but, when PVA is added for blends’ formation, the morphology is converted from smooth to micro-phase separated, which inferred the change in miscibility of two types of polymers from good to a certain value of miscibility. In these formulations, the MMT (reinforcement) was consistently dispersed in the matrix of film [42]. This proved its high compatibility in the polymer matrix, as there were no gaps or cracks in the formulations.

### 3.4. EDX Analysis 

**Figure 4:**
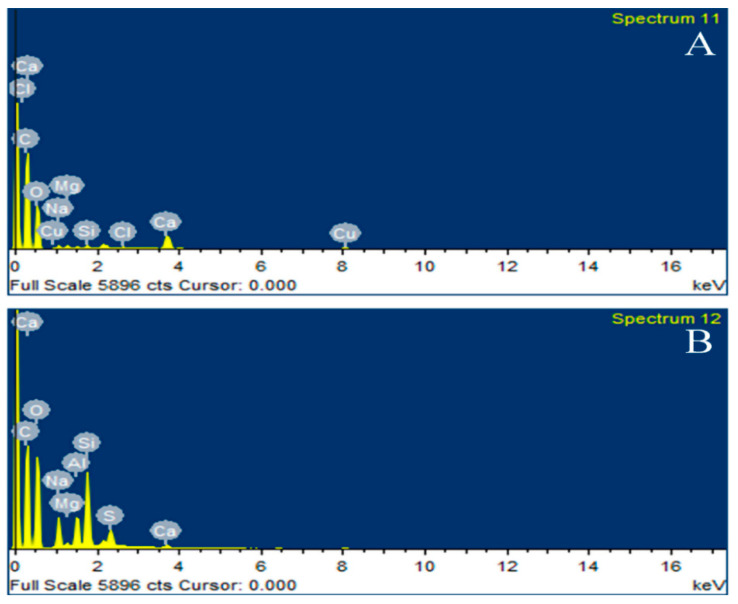
EDX analysis of (**A**) S50P50, (**B**) S50P50D15(3).

**Figure 5:**
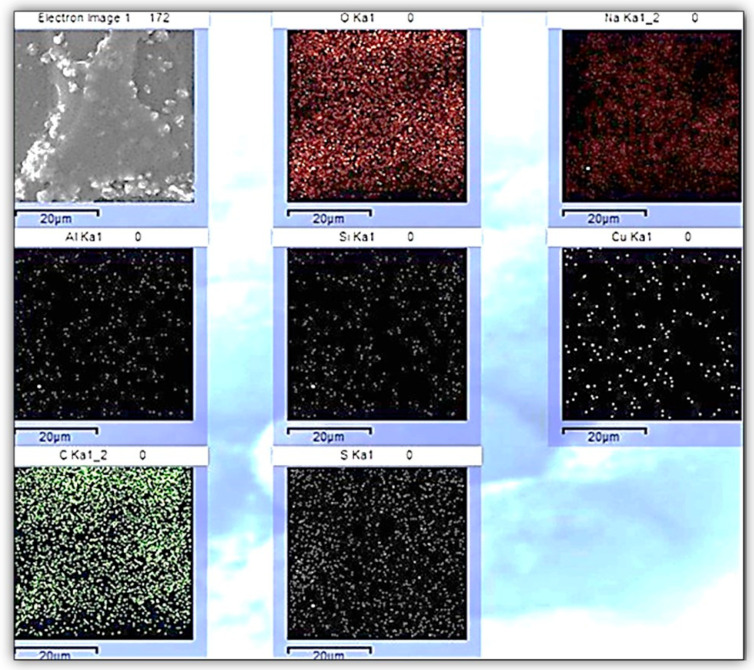
EDX Mapping of S50P50D15.

Furthermore, using the SEM technique, the purity of the S50P50(3) and S50P50D15(3) mixing component and elemental composition were determined by EDX analysis (Figure 4A,B). EDX with a mapping technique was also applied for S50P50(3) (data not shown) and S50P50D15(3) (Figure 5). 

Figure 4A represents the elemental composition of S50P50(3). Peaks of C, O, Na, Ca, Mg, and Si are displayed. The intense peaks of C, O, and Na defined SA and PVA in greater proportion, while low intensity peaks of Ca, Mg, Si, and Na indicated the presence of MMT. A peak of chlorine in AP50 revealed Cl impurity in it. 

Figure 4B shows elemental composition of S50P50D15(3), here we can notice the peak of sulphur, confirming the presence of CTX.

Figure 5 shows EDX mapping analysis of S50P50D15. It shows the homogeneity of the synthesized material. It also shows the uniform dispersion and distribution of the drug-containing Sulphur atom. Similarly, the Si atom which is found in nano-clay is also uniformly dispersed in the polymeric matrix. 

### 3.5. Swelling Study

**Figure 6:**
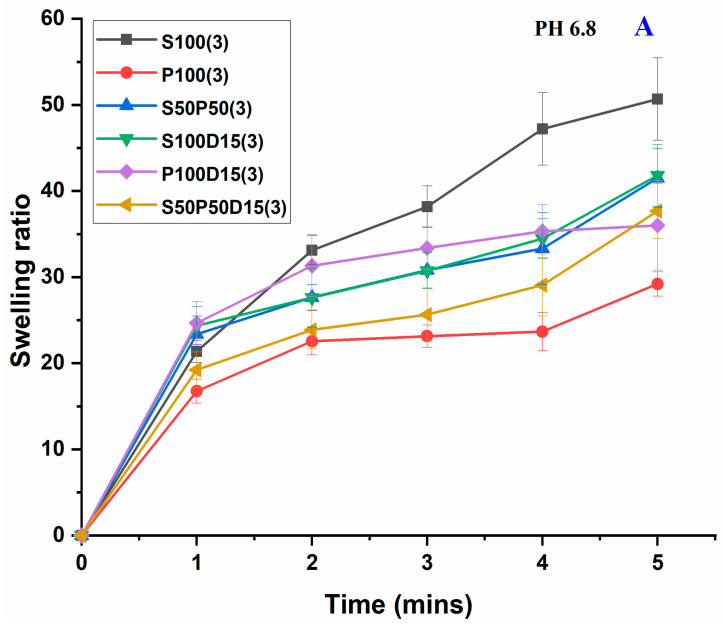

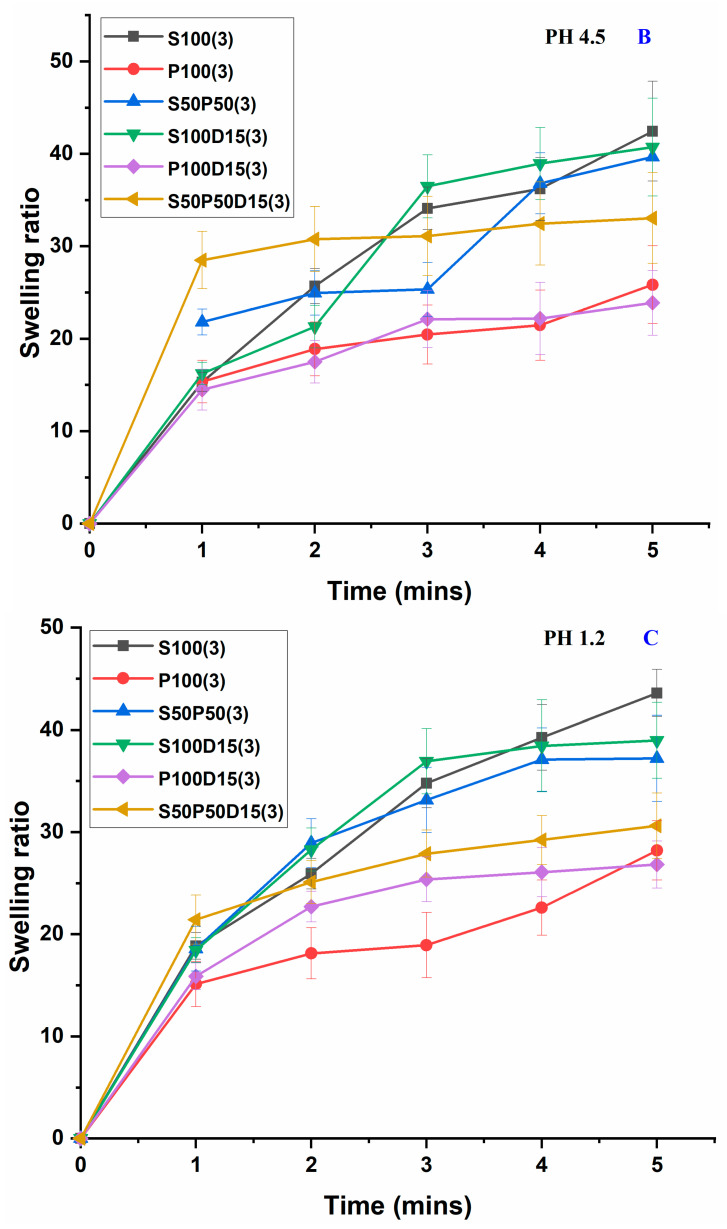
Swelling ratio of drugloaded (and unloaded) SA/PVA-MMT clay-reinforced nanocomposite films in (**A**) 1× PBS, pH 6.8, (**B**) NaOAc solution, pH 4.5, and (**C**) HCl solution, pH 1.2.

The swelling behavior of clay-reinforced nanocomposite formulations (i.e., S100(3), P100(3), S50P50(3), S100D15(3), P100D15(3), and S50P50D15(3)) was studied in buffers at pH 6.8 (1× PBS), 4.5 (NaOAc), and 1.2 (HCl) (Figure 6A, 6B and 6C, respectively). The swelling action of such films is controlled by the nature of the polymer, extent of cross-linking, and polymer–solvent aptitude [39,43].

The polymer nanocomposites showed in their swelling behavior (Figure 6A–C). The formulations S100(3), P100(3), and S50P50(3) were formulated with a constant amount of clay but a varied number of polymers. A decrease in swelling with an increase in PVA concentration in the prepared films can be observed. The least swelling was seen in P100(3) which is made up of 100% PVA and 0% SA. In contrast, the formulation S100(3) showed maximum swelling ratio, which means that SA is responsible for this effect. An increase in SA concentration increased swelling due to the hydrophilic behavior of SA which elevated the swelling aptitude [43].

The maximum swelling action was seen in a buffer of pH 6.8 (Figure 6A), compared to pH 4.5 (Figure 6B) and pH 1.2 (Figure 6C). Thus, an increase in the swelling ratio could be observed with an increase in the pH of the buffers. This can be explained by the fact that in buffers of low pH, the carboxylic group of SA is strongly protonated resulting in maximum H-bonding among hydroxyl of carboxylic groups and H^+^ ions of water. This blocks the water absorption. However, at pH 6.8, which is close to neutral, the hydrophilic behavior of films has increased. Thereby, an increase in the swelling ratio with an increase in the pH of the buffer, due to de-protonation of the carboxylic group on SA, produced a strong swelling action by electrostatic forces of repulsion on the negatively charged group of SA and PVA. Our findings agree with those in the literature [41,42,43]. Additionally, the decrease in swelling ratio is due to the formation of the hydrogen bonding network of MMT with the polymers, which enhances the surface coarseness of films and closed the water penetration corridor resulting in a decrease in water compassion of the polymer films [42]. Also, the Si-O-Si group of MMT is the basic functional group which is responsible for communication to drug and polymer. The addition of clay produces Si-O-Si groups for interaction and creates more cross-linking in the structure, which increases the number of pores but decreases the pore size, thereby causing decreases in the swelling ratio [42]. Eventually, MMT is less hydrophilic than SA and PVA, so its merger lowered the swelling ratio. 

In this series, the lowest swelling capacity was shown by drug-loaded nanocomposites, which showed a decrease in swelling behavior, because of the cross-linked compactness of the polymer network that lessens free volume resulting in decrease of water diffusion. 

### 3.6. Erosion Studies

All the six formulations of clay reinforced polymer nanocomposites were studied for erosion behavior in buffers at pH 6.8 (1× PBS), 4.5 (NaOAc), and 1.2 (HCl) (Table 3). The data inferred those formulations having higher concentrations of SA showed more erosion relative to formulations containing more PVA. 

At pH 6.8, S100(3) was the formulation that showed the maximum value of erosion (91.7% ± 0.017), while no erosion was observed in P100(3). Similar erosion behavior (*p* > 0.05) was found in S100D15(3), the formulation of which was slightly different (*p* < 0.05) from S50P50D15(3) but highly different (*p* < 0.01) from P100(3), S50P50(3), and P100D15(3). 

At pH 4.5, S100D15(3) was the formulation that showed the maximum value of erosion (82.6% ± 0.001). Similar erosion behavior (*p* > 0.05) was found in S50P50(3), the formulation of which was slightly different (*p* < 0.05) from P100D15(3) and S50P50D15(3) but highly different (*p* < 0.01) from P100(3).

At pH 1.2, S100D15(3) was the formulation that showed the maximum value of erosion (84.7% ± 0.00). Similar erosion behavior (*p* > 0.05) was found in S100(3), the formulation of which was slightly different (*p* < 0.05) from S50P50(3) and S50P50D15(3) but highly different (*p* < 0.01) from P100(3) and P100D15(3).

Inter-analyses shows that the erosion behavior of S100(3) at pH 1.2 and pH 4.5 is statistically similar (*p* > 0.05) to S100D15(3) at pH 1.2 and pH 4.5, strongly suggesting that the drug has no additive or synergic effect on the erosion behavior mainly caused by SA. At pH 6.8, the erosion of S100(3) and S100D15(3) is significantly higher due to the absence of PVA in the formulation which, as previously shown, led to deswelling. 

Further analyses show that pH-independent erosion behavior roughly follows this order: S100(3) > S100D15(3) > S50P50D15(3) > S50P50(3) > P100D15(3) > P100(3), which strongly suggests that this behavior can be attributed to SA and not to PVA. It also can be explained by the increase in number of pores and the decrease in size of pores upon addition of clay; this produces more channels for release of water which led to a boost in deswelling and erosion [31,43].

### 3.7. Drug Permeation Study

**Figure 7:**
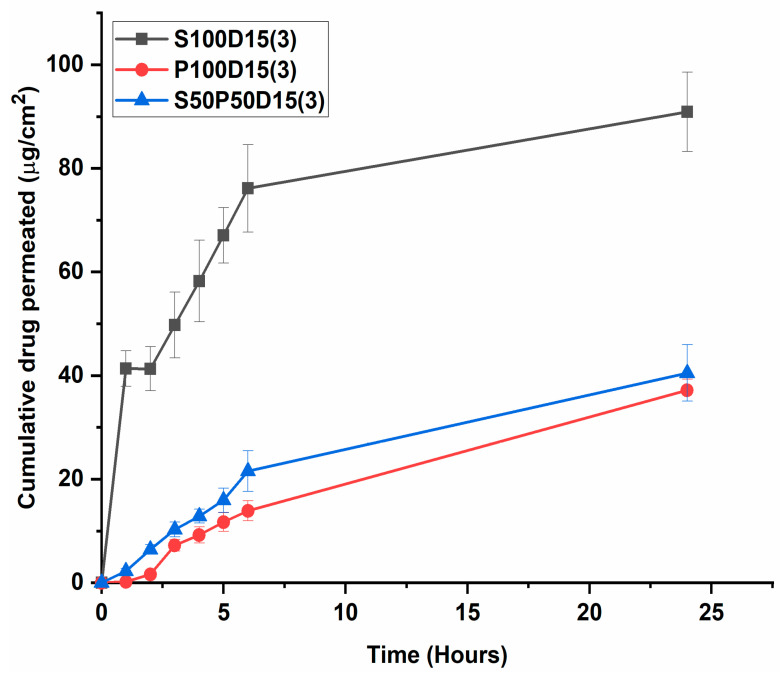
Cumulative drug permeated from drug loaded SA/PVA-MMT clay-reinforced nanocomposite films at pH 6.8 in 1× PBS.

The cumulative drug permeated through polymer nanocomposites films, i.e., the calculation of the approximate rate of drug discharge, was carried out from S100D15(3), P100D15(3), and S50P50D15(3) formulations through the rat skin in 24 h. This ex vivo study was performed with a time break of 1, 2, 3, 4, 5, 6, and 24 h at pH 6.8 in 1× PBS using Franz diffusion cell (Figure 7). The dilution method was used to plot the standard calibration curve at pH 6.8 in 1× PBS.

Previous studies showed that as the concentration of the drug is increased, the cumulative drug permeated is decreased. The films with the highest amount of drug gave the lowest values of the permeation [33,34]. We found similar findings (data not shown). Interestingly, S100D15(3) is the formulation that showed the maximum drug release in this series (*p* < 0.01), most likely due to the hydrophilic nature of SA [18]. The data showed a decrease (*p* < 0.05) in drug release with an increase in PVA concentration.

### 3.8. In Vitro Drug Release Behavior 

**Figure 8:**
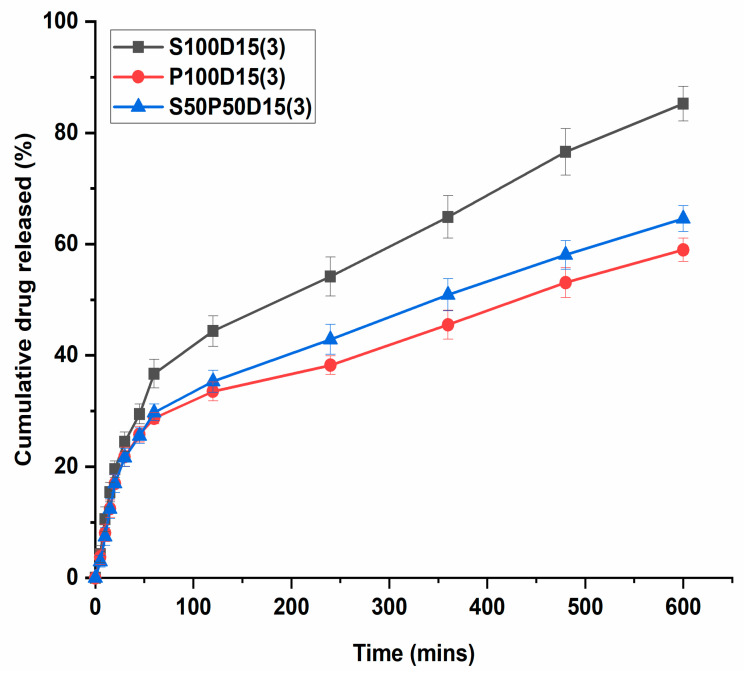
In vitro drug release behavior from drug-loaded SA/PVA-MMT clay-reinforced nanocomposite films at pH 6.8 in 1× PBS.

The drug release behavior was carried out from S100D15(3), P100D15(3), and S50P50D15(3) formulations at pH 6.8 in 1× PBS, and the percentage of the permeated drug discharge was estimated with a time break of 5, 10, 15, 20, 30, 45, 60, 120, 240, 360, 480, and 600 min (Figure 8). 

Likewise in the drug permeation study, the drug-loaded formulations showed an outstanding, continuous discharge of drug for 600 min, and S100D15(3) is the formulation that showed the best cumulative drug release (*p* < 0.01) compared to P100D15(3) and S50P50D15(3) (Figure 8). For all the formulations, the results revealed a bi-step discharge mode with early burst and a quick liberation chased by continued the drug discharge of devised nanoparticles. The dissolution studies of drug-loaded polymer nanocomposites elicited sustained drug delivery. The early quick discharge of drug may also be due to the presence of the drug on the film face [20,21].

The progressive decrease in the magnitude of drug released from the film (fall in the speed of drug discharge) can be attributed to [20,21,22,28,29,34] (i) the progressive increase in concentration of the eroded polymers due to which the system became more viscous. Indeed, the drug/polymer ratio is another factor which controls the drug release. Thereby, the drug release decreases with an increase in drug/polymer ratio. The high drug concentration results in the formation of a compact network of the polymer due to maximum cross-linking, and the small free volume in dense polymers lowers the diffusion of water molecules; (ii) the entrapment of the drug inside the polymeric film; (iii) the contribution of many OH and carboxyl groups of the SA in the polymerization of PVA and development of H-bond between PVA and SA. 

In contrast, its burst release of the drug from the films is linked to [22,28,39,43] (i) the increase in swelling along with the drug dissolution due to a weak constancy in aqueous medium; Therefore, the easier the diffusion of buffer into the films, the quicker the drug discharge; (ii) when the drug concentration is low, it forms a loose structure in the polymeric films, and large pore volume is formed; this also boosts the drug discharge. 

## 4. Conclusions

Various formulations of SA/PVA clay-reinforced polymer nanocomposites were synthesized by solution casting technique, in which CTX was (or not) encapsulated. The prepared formulations were characterized by physical techniques followed by pharmaceutical studies. TGA data revealed that SA is thermally more stable than PVA, since the maximum residue (23.8%) was left by S100D15(3) formulation, and the least stability was observed for P100D15(3) by leaving a minimal residue (4.02%). Moreover, the addition of drug increases the thermal stability of drug-loaded nanocomposites, which is inferred by the fact that the S50P50(3) without CTX sample left only 16.1% residue whereas the CTX-loaded sample S50P50D15(3) left 20.4% residue. XRD investigation exhibited the amorphous character of SA and the crystalline behavior of PVA. Sharp peaks with reduced 2θ and higher d-spacing values were observed for MMT nanoclay. The drug-loaded formulations are amorphous in nature and showed broad peaks. SEM and EDX analysis revealed the uniform distribution and purity of the mixing components. Surface morphology revealed the presence of voids in the films generating a highly porous structure, which is responsible for the hydrophilic behavior of films. The average particle size of the formulations ranged from 300 nm to 500 nm. The maximum swelling and erosion were observed at pH 6.8. However, all those formulations having the highest concentration of SA revealed the highest values of swelling and erosion. According to dissolution and permeation data, an increase in SA concentration enhanced the drug permeability and dissolution. Eventually, we conclude that SA/PVA nanocomposite films reinforced with MMT clay could be applied for the targeted, controlled, and sustained release of CTX in wound care (as wound dressing material). 

## Figures and Tables

**Table 1:** CTX-loaded (or unloaded) SA/PVA nanocomposites reinforced with MMT nanoclay (*n* = 6). All the values are expressed in g.

#	SA (S)	PVA (P)	CTX Drug (D)	Nanoclay MMT (M)	Glycerol	Code Formulation
1	100	0	0	3	50	S100(3)
2	0	100	0	3	50	P100(3)
3	50	50	0	3	50	S50P50(3)
4	100	0	15	3	50	S100D15(3)
5	0	100	15	3	50	P100D15(3)
6	50	50	15	3	50	S50P50D15(3)

**Table 2:** Percentage of mass loss in drug loaded (and unloaded) SA/PVA-MMT clay reinforced nanocomposite films at different temperatures.

#	Formulation Code SA/PVA Ratio	Mass Loss (%) 120 °C	Mass Loss (%) 200 °C	Mass Loss (%) 400 °C	% Weight Residue at 600 °C
1.	S50P50(3)	18.6	34.1	79.5	16.1
2.	S100D15(3)	23.8	43.3	77.3	23.8
3.	P100D15(3)	13.0	34.5	81.0	4.02
4.	S50P50D15(3)	19.1	37.2	74.8	20.4

**Table 3:** Percentage of erosion of drug loaded (and unloaded) SA/PVA-MMT clay-reinforced nanocomposite.

Formulation Codes	Erosion (% ± SD)		
	pH 6.8	pH 4.5	pH 1.2
S100(3)	91.7 ± 0.017 ^a^	80.1 ± 0.003 ^b^	77.8 ± 0.015 ^b^
P100(3)	0 ^d^	58.9 ± 0.002 ^d^	46.0 ± 0.005 ^e^
S50P50(3)	69.4 ± 0.001 ^c^	78.1 ± 0.024 ^b^	73.0 ± 0.0037 ^c^
S100D15(3)	89.0 ± 0.001 ^a^	82.6 ± 0.001 ^b^	84.7 ± 0.00 ^b^
P100D15(3)	71.57 ± 0.08 ^c^	72.6 ± 0.001 ^c^	59.6 ± 0.014 ^d^
S50P50D15(3)	81.4 ± 0.024 ^b^	76.2 ± 0.003 ^c^	72.8 ± 0.026 ^c^

Films in 1× PBS—pH 6.8; NaOAc solution—pH 4.5; (C) HCl solution—pH 1.2. Same letters mean no statistical significance; different letters mean statistical difference.

## Data Availability

The data presented in this study are available on request from the corresponding author.

## References

[1] Kamel S., Ali N., Jahangir K., Shah S.M., El-Gendy A.A. (2008). Pharmaceutical significance of cellulose: A Review. Express Polym. Lett..

[2] Chen Y., Zhang Y., Wang F.J., Meng W.W., Yang X.L., Jiang J.X., Tan H.M., Zheng Y.F. (2016). Preparation of the porous carboxymethyl chitosan grafted poly (acrylic acid) superabsorbent by solvent precipitation and its application as a hemostatic wound dressing. Mater. Sci. Eng. C.

[3] Alavijeh M.S., Chishty M., Qaiser M.Z., Palmer A.M. (2005). Drug metabolism and pharmacokinetics, the blood-brain barrier, and central nervous system drug discovery. NeuroRx.

[4] Ray S., Banerjee S., Maiti S., Laha B., Barik S., Sa B., Bhattacharyya U.K. (2010). Novel interpenetrating network microspheres of xanthan gum-poly(vinyl alcohol) for the delivery of diclofenac sodium to the intestine—In vitro and in vivo evaluation. Drug Deliv..

[5] Ferraz M.P., Monteiro F.J., Manuel C.M. (2004). Hydroxyapatite nanoparticles: A review of preparation methodologies. J. Appl. Biomater. Biomech..

[6] Chan W.C. (2006). Bionanotechnology progress and advances. Biol. Blood Marrow Transpl..

[7] Menaa F. (2013). When Pharma Meets Nano or The Emerging Era of Nano-Pharmaceutical. Pharm. Anal. Acta.

[8] Salamanca C.H., Barrera-Ocampo A., Lasso J.C., Camacho N., Yarce C.J. (2018). Franz Diffusion Cell Approach for Pre-Formulation Characterisation of Ketoprofen Semi-Solid Dosage Forms. Pharmaceutics.

[9] Iliescu R.I., Andronescu E., Voicu G., Ficai A., Covaliu C.I. (2011). Hybrid materials based on montmorillonite and citostatic drugs. Prep. Charact. Ceftriaxone Sodium Encapsulated Chitosan Nanopart..

[10] Suresh R., Borkar S.N., Sawant V.A., Shende V.S., Dimble S.K. (2010). Nanoclay drug delivery system. Int. J. Pharm. Sci. Nanotechnol..

[11] Winey K.I., Vaia R.A. (2007). Polymer nanocomposites. MRS Bull..

[12] Rong M.Z., Zhang M.Q., Ruan W.H. (2006). Surface modification of nanoscale fillers for improving properties of polymer nanocomposites: A review. Mater. Sci. Technol..

[13] Jana S., Sen K.K., Gandhi A. (2016). Alginate Based Nanocarriers for Drug Delivery Applications. Curr. Pharm. Des..

[14] Niculescu A.G., Grumezescu A.M. (2022). Applications of Chitosan-Alginate-Based Nanoparticles-An Up-to-Date Review. Nanomaterials.

[15] Weng L., Zhang X., Fan W., Lu Y. (2021). Development of the inorganic nanoparticles reinforced alginate-based hybrid fiber for wound care and healing. J. Appl. Polym. Sci..

[16] Krebs M.D., Salter E., Chen E., Sutter K.A., Alsberg E. (2010). Calcium phosphate-DNA nanoparticle gene delivery from alginate hydrogels induces in vivo osteogenesis. J. Biomed. Mater. Res. A.

[17] Wang F., Yang S., Yuan J., Gao Q., Huang C. (2016). Effective method of chitosan-coated alginate nanoparticles for target drug delivery applications. J. Biomater. Appl..

[18] Tuğcu-Demiröz F., Acartürk F., Takka S., Konuş-Boyunağa Ö. (2007). Evaluation of alginate based mesalazine tablets for intestinal drug delivery. Eur. J. Pharm. Biopharm..

[19] Kim J.O., Park J.K., Kim J.H., Jin S.G., Yong C.S., Li D.X., Choi J.Y., Woo J.S., Yoo B.K., Lyoo W.S. (2008). Development of polyvinyl alcohol–sodium alginate gel-matrix-based wound dressing system containing nitrofurazone. Int. J. Pharm..

[20] Şanlı O., Ay N., Işıklan N. (2007). Release characteristics of diclofenac sodium from poly (vinyl alcohol)/sodium alginate and poly (vinyl alcohol)-grafted-poly (acrylamide)/sodium alginate blend beads. Eur. J. Pharm. Biopharm..

[21] Han X., Huo P., Ding Z., Kumar P., Liu B. (2019). Preparation of Lutein-Loaded PVA/Sodium Alginate Nanofibers and Investigation of Its Release Behavior. Pharmaceutics.

[22] Razzaq A., Khan Z., Saeed A., Shah K., Khan N., Menaa B., Iqbal H., Menaa F. (2021). Development of Cephradine-Loaded Gelatin/Polyvinyl Alcohol Electrospun Nanofibers for Effective Diabetic Wound Healing: In Vitro and In Vivo Assessments. Pharmaceutics.

[23] Khan B.A., Ullah S., Khan M.K., Uzair B., Menaa F., Braga V.A. (2020). Fabrication, Physical Characterizations, and In Vitro, In Vivo Evaluation of Ginger Extract-Loaded Gelatin/Poly(Vinyl Alcohol) Hydrogel Films Against Burn Wound Healing in Animal Model. AAPS Pharm. Sci. Tech..

[24] Tewabe A., Marew T., Birhanu G. (2021). The contribution of nano-based strategies in overcoming ceftriaxone resistance: A literature review. Pharmacol. Res. Perspect..

[25] Alshubaily F.A., Al-Zahrani M.H. (2019). Appliance of fungal chitosan/ceftriaxone nano-composite to strengthen and sustain their antimicrobial potentiality against drug resistant bacteria. Int. J. Biol. Macromol..

[26] Khan Z.U., Razzaq A., Khan A., Rehman N.U., Khan H., Khan T., Khan A.U., Althobaiti N.A., Menaa F., Iqbal H. (2022). Physicochemical Characterizations and Pharmacokinetic Evaluation of Pentazocine Solid Lipid Nanoparticles against Inflammatory Pain Model. Pharmaceutics.

[27] Wang X., Du Y., Luo J., Lin B., Kennedy J.F. (2007). Chitosan/organic rectorite nanocomposite films: Structure, characteristic and drug delivery behaviour. Carbohydr. Polym..

[28] Iqbal H., Khan B.A., Khan Z.U., Razzaq A., Khan N.U., Menaa B., Menaa F. (2020). Fabrication, physical characterizations and in vitro antibacterial activity of cefadroxil-loaded chitosan/poly(vinyl alcohol) nanofibers against Staphylococcus aureus clinical isolates. Int. J. Biol. Macromol..

[29] Zafar N., Uzair B., Niazi M.B.K., Menaa F., Samin G., Khan B.A., Iqbal H., Menaa B. (2021). Green Synthesis of Ciprofloxacin-Loaded Cerium Oxide/Chitosan Nanocarrier and its Activity Against MRSA-Induced Mastitis. J. Pharm. Sci..

[30] Cao Z., Yang Q., Fan C., Liu L., Liao L. (2015). Biocompatible, ionic strength sensitive, double network hydrogel based on chitosan and an oligo (trimethylene carbonate)–poly (ethylene glycol)–oligo (trimethylene carbonate) triblock copolymer. Appl. Polym. Sci..

[31] Lamoudi L., Chaumeil J.C., Daoud K. (2016). Swelling, erosion and drug release characteristics of sodium diclofenac from heterogeneous matrix tablets. Drug Deliv. Sci. Technol..

[32] Ethiraj R., Thiruvengadam E., Sampath V.S., Vahid A., Raj J. (2014). Development and Validation of Stability Indicating Spectroscopic Method for Content Analysis of Ceftriaxone Sodium in Pharmaceuticals. Int. Sch. Res. Not..

[33] Ng S.F., Rouse J., Sanderson D., Eccleston G. (2010). A comparative study of transmembrane diffusion and permeation of ibuprofen across synthetic membranes using Franz diffusion cells. Pharm. Pharmacol. Lett..

[34] Khan M.K., Khan B.A., Uzair B., Iram Niaz S., Khan H., Hosny K.M., Menaa F. (2021). Development of Chitosan-Based Nanoemulsion Gel Containing Microbial Secondary Metabolite with Effective Antifungal Activity: In vitro and in vivo Characterizations. Int. J. Nanomed..

[35] Soares J.D., Santos J.E., Chierice G.O., Cavalheiro E.T. (2004). Thermal behaviour of alginic acid and its sodium salt. Eclet. Quim. Sao Paulo.

[36] Ahmad M.B., Gharayebi Y., Salit M.S., Hussein M.Z., Shameli K. (2011). Comparison of in situ polymerization and solution-dispersion techniques in the preparation of polyimide/montmorillonite (MMT) nanocomposites. Int. J. Mol. Sci.

[37] Nešić A., Onjia A., Davidović S., Dimitrijević S., Errico M.E., Santagata G., Malinconico M. (2017). Design of pectin-sodium alginate-based films for potential healthcare application: Study of chemico-physical interactions between the components of films and assessment of their antimicrobial activity. Carbohydr. Polym..

[38] Sabaa M.W., Abdallah H.M., Mohamed N.A., Mohamed R.R. (2015). Synthesis, characterization, and application of biodegradable crosslinked carboxymethyl chitosan/poly (vinyl alcohol) clay nanocomposites. Mater. Sci. Eng. C.

[39] Yadav M., Rhee K.Y. (2012). Superabsorbent nanocomposite (alginate-g-PAMPS/MMT) Synthesis, characterization, and swelling behavior. Carbohydr. Polym..

[40] Navarchian A.H., Joulazadeh M., Mousazadeh S. (2013). Application of the taguchi approach to investigate the effects of clay content and saponification parameters on the tensile properties of poly (vinyl alcohol)/clay nanocomposites. J. Vinyl Addit. Technol..

[41] Youdhestar Mahar F.K., Das G., Tajammul A., Ahmed F., Khatri M., Khan S., Khatri Z. (2022). Fabrication of Ceftriaxone-Loaded Cellulose Acetate and Polyvinyl Alcohol Nanofibers and Their Antibacterial Evaluation. Antibiotics.

[42] Jung H.M., Lee E.M., Ji B.C., Deng Y., Yun J.D., Yeum J.H. (2007). Poly (vinyl acetate)/poly (vinyl alcohol)/montmorillonite nanocomposite microspheres prepared by suspension polymerization and saponification. Colloid Polym. Sci..

[43] Calik M.K., Ozdemir M. (2016). Synthesis, characterization and, swelling behavior of semi IPN nanocomposite hydrogels of alginate with poly (N isopropylacrylamide) crosslinked by nanoclay. Appl. Polym. Sci..

